# Computational Analysis of the Interaction Energies between Amino Acid Residues of the Measles Virus Hemagglutinin and Its Receptors

**DOI:** 10.3390/v10050236

**Published:** 2018-05-03

**Authors:** Fengqi Xu, Shigenori Tanaka, Hirofumi Watanabe, Yasuhiro Shimane, Misako Iwasawa, Kazue Ohishi, Tadashi Maruyama

**Affiliations:** 1 Department of Computational Science, Graduate School of System Informatics, Kobe University, 1-1, Rokkodai, Nada-ku, Kobe, Hyogo 657-8501, Japan; xu@eniac.scitec.kobe-u.ac.jp; 2 Education Center on Computational Science and Engineering, Kobe University, 7-1-48, Minatojima-minamimachi, Chuo-ku, Kobe 650-0047, Japan; hirofumi.watanabe@port.kobe-u.ac.jp; 3 Center for Marine Biosciences, Japan Agency for Marine-Earth Science and Technology, 2-15, Natsushima, Yokosuka, Kanagawa 237-0061, Japan; yshimane@jamstec.go.jp (Y.S.); pee02660@nifty.ne.jp (T.M.); 4 Center for Earth Information Science and Technology, Japan Agency for Marine-Earth Science and Technology, 3173-25, Showa-machi, Kanazawa-ku, Yokohama, Kanagawa 236-0001, Japan; misa0130@gamma.ocn.ne.jp; 5 Faculty of Engineering, Tokyo Polytechnic University, 1583, Iiyama, Atsugi, Kanagawa 243-0297, Japan; cie20910@syd.odn.ne.jp

**Keywords:** measles virus, hemagglutinin, receptors, molecular recognition, fragment molecular orbital (FMO) method, IFIE (inter-fragment interaction energy)

## Abstract

Measles virus (MV) causes an acute and highly devastating contagious disease in humans. Employing the crystal structures of three human receptors, signaling lymphocyte-activation molecule (SLAM), CD46, and Nectin-4, in complex with the measles virus hemagglutinin (MVH), we elucidated computationally the details of binding energies between the amino acid residues of MVH and those of the receptors with an *ab initio* fragment molecular orbital (FMO) method. The calculated inter-fragment interaction energies (IFIEs) revealed a number of significantly interacting amino acid residues of MVH that played essential roles in binding to the receptors. As predicted from previously reported experiments, some important amino-acid residues of MVH were shown to be common but others were specific to interactions with the three receptors. Particularly, some of the (non-polar) hydrophobic residues of MVH were found to be attractively interacting with multiple receptors, thus indicating the importance of the hydrophobic pocket for intermolecular interactions (especially in the case of Nectin-4). In contrast, the electrostatic interactions tended to be used for specific molecular recognition. Furthermore, we carried out FMO calculations for *in silico* experiments of amino acid mutations, finding reasonable agreements with virological experiments concerning the substitution effect of residues. Thus, the present study demonstrates that the electron-correlated FMO method is a powerful tool to search exhaustively for amino acid residues that contribute to interactions with receptor molecules. It is also applicable for designing inhibitors of MVH and engineered MVs for cancer therapy.

## 1. Introduction

Measles virus (MV) belonging to the genus *Morbillivirus* of the family Paramyxoviridae is a causative agent of measles in humans. Measles is an acute and highly contagious disease that often induces immunosuppression. Measles is still a serious disease in children, especially in developing countries [1]. As MV is serologically monotypic, effective vaccination programs are expected to achieve global eradication. The extensive vaccination campaign promoted by the World Health Organization (WHO) has dramatically decreased the number of measles cases. However, as the occurrence of diseases and deaths is still recorded especially in Asia and Africa, the risk of MV transmission needs to be considered in the current globalized world.

Cellular receptor molecules on host cells are needed for viral entry. They determine the virus tropism of cells and tissues, and the host specificities of the virus; they are also deeply related to pathogenesis. For MV, three distinct receptor molecules, signaling lymphocyte-activation molecule (SLAM), Nectin-4, and CD46, have been identified. SLAM is a principal receptor for virus entry, and is expressed selectively in immune cells such as monocytes, dendritic cells and activated T and B cells [2,3]. Nectin-4 is expressed at the adherens junction of epithelial cells, and is used for virus release from the human body [4,5,6]. In addition to SLAM and Nectin-4, only some vaccine MV strains can use CD46 as a receptor, which is a complement-regulatory protein expressed in all nucleated human cells [7,8].

MV possesses two viral envelope glycoproteins, hemagglutinin (H) and fusion (F) proteins, on its surface. H binds to the receptors and then initiates fusion between the F protein and host cell membrane in the initial step of MV invasion [9]. The H proteins form homophilic dimers, and each H protein contains the stalk and head domains. The head domain exhibits six-bladed β-propeller folds [10]. Recently the crystal structures of respective complexes of the measles virus H protein (MVH) and the three receptors mentioned above have been determined [11,12,13]. These studies have demonstrated that MVH uses the same β4-β5 groove to bind with all three receptors. On the other hand, there are considerable differences among the structures of the three receptors binding to the MVH [11,12,13].

It is essential to elucidate the details of intermolecular interactions quantitatively in order to understand the molecular recognition mechanisms involved in the complexes of MVH and the respective receptors. The interaction between MVH and SLAM has been analyzed by using the docking analysis and expressed in terms of docking scores [14]. However, the interaction energies of respective amino acid residues of the receptor and MVH remain to be studied more accurately. Here, we conducted fine computational analyses of the interactions between the H protein and the respective three receptors by *ab initio* fragment molecular orbital (FMO) method [15,16,17], which is a quantum-chemical calculation method to study the electronic states and the interactions of large biomolecules with high accuracy and reduced computational costs. This comprehensive calculation of the affinity between amino acid residues on the viral H and the host receptors enables the specification of critical amino acid residues for binding. In the present study, we analyzed theoretically and predicted the effects of replacing these important residues with other amino acids on binding affinity in comparison with the reported virological experiments. These results also provide important insights into antiviral drug design and the quality control of vaccines and live MV-based vectors for cancer therapy [18,19,20,21].

The present study aims to illustrate that our *ab initio* FMO-inter-fragment interaction energy (IFIE) analysis can provide comprehensive and quantitative descriptions concerning the molecular recognition between the three receptors and the wild-type or mutant MVH in a manner consistent with the experimental results. After examining the advantage of computational approach to elicit exhaustive information on the interactions at residue level, we will provide a perspective for its potential medical applications. The important point to be considered is that the three receptors bind to MVH at somewhat overlapping interfaces, thus indicating some similarities and differences in the mechanism of their molecular recognition (or binding energy) which can be utilized for artificially controlling the (relative) strength of respective binding affinity. Thus, the present quantum-chemical approach can provide a more dependable theoretical tool than conventional docking simulations [14].

## 2. Materials and Methods

**Figure 1:**
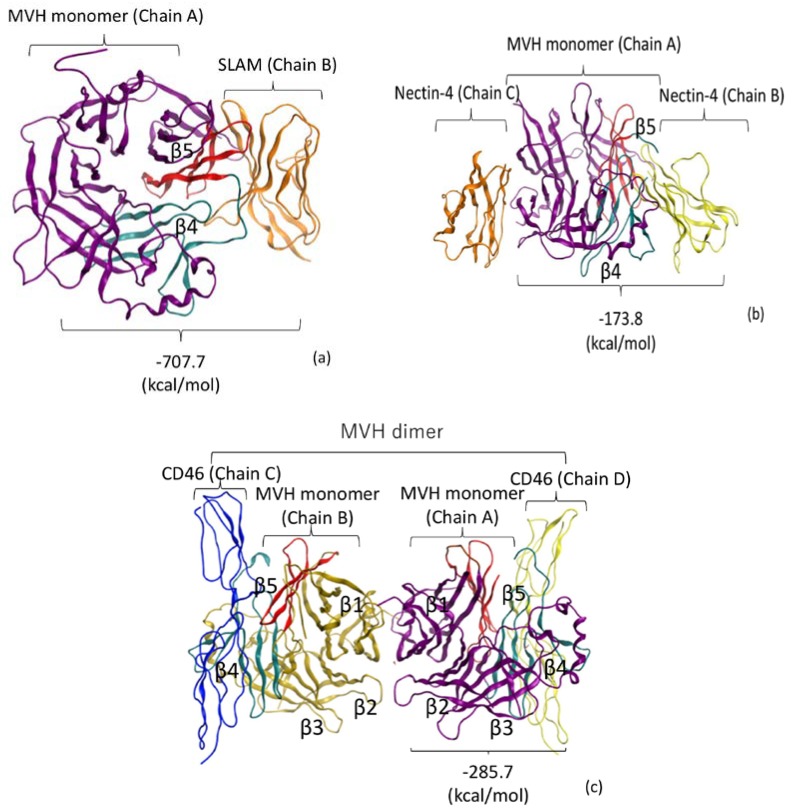
Crystal structures [11,12,13] of measles virus H protein (MVH)-receptor complexes employed for the fragment molecular orbital (FMO) calculations, where the β4 and β5 strand regions in MVH are depicted in cyan and red colors, respectively, along with the calculated values of inter-fragment interaction energies (IFIE) sums for the MVH-receptor interactions. (**a**) Complex of signaling lymphocyte-activation molecule (SLAM) and MVH, where Chain B (SLAM) is complexed with Chain A (MVH). (**b**) Complex of Nectin-4 and MVH. Two receptor chains, Chain B and Chain C, are complexed with MVH (Chain A) at different interfaces. The binding of Chain C is regarded as an artifact because of crystal packing [13]. (**c**) Dimeric complex of CD46 and MVH. Chain C and Chain D of the CD46 receptor are complexed with Chain B and Chain A of MVH, respectively, where the β1–β5 regions in MVH are designated.

To analyze the molecular interactions between MVH and three human receptors, SLAM, Nectin-4, and CD46, we retrieved the crystal structures of their complexes from the Protein Data Bank (PDB entries: 3ALZ for SLAM, 4GJT for Nectin-4, and 3INB for CD46) for the FMO calculations (Figure 1). In these structures, the complex of MVH–SLAM had been generated as MVH monomer (Chain A) and one SLAM molecule (Chain B) (Figure 1a). The complex of MVH–Nectin-4 had been constructed as MVH monomer (Chain A) and two Nectin-4 molecules (Chains B and C) (Figure 1b). Because the binding between the Chain A and the Chain C had been reported as an artifact due to crystal packing during complex crystallization [13], we did not consider it in the present study. The complex of MVH–CD46 had been generated as MVH dimer (Chains A and B) and two CD46 molecules (Chains C and D) (Figure 1c). In the present computational analysis, the complex of Chain A and Chain D was analyzed according to the crystallographic study by Santiago et al. [11].

In this study, molecular interactions were analyzed by the FMO method with electron-correlated MP2/6-31G* scheme using the software ABINIT-MP [16,17]. Preparations of the complex structures used for FMO calculations were carried out through molecular modeling by the Molecular Operating Environment (MOE, CCG Inc., Montreal, QC, Canada) [22]. After complementation of missing atoms (for capping the missing residues) and addition of hydrogen atoms, the positions of the added atoms were energetically optimized with the AMBER force field [22]. The inter-fragment interaction energies (IFIEs) [17] which are obtained as effective interactions between fragments (residues) in the quantum-chemical FMO calculations then provide useful information about the important amino-acid pairs between MVH and the receptors. For example, the negative value of IFIE indicates an attractive interaction between the two fragments representing an amino acid pair. The summation of all the IFIEs between residue pairs of MVH and receptor gives the binding energy. In addition to the wild-type MVHs, we also performed FMO calculations for several mutants that have been studied experimentally or have been predicted to modify affinity; substitutions of pertinent amino-acid residues were made with the aid of MOE software, along with structural (energetic) optimization for the mutants. Note that the present FMO calculations were carried out without taking into account the solvation effects, which may lead to some overestimation of electrostatic interactions between charged residues, whereas the order of importance of residues in molecular recognition would not be affected significantly [17]. Considering the screening effects in the Coulombic interactions, the IFIEs between fragments whose distances are within 5 Å have been taken into account in the present FMO analysis. The summation of IFIEs under this restriction is referred to as the “IFIE sum” hereafter. The usefulness of the FMO-IFIE analysis has also been successfully verified in the case of the influenza virus hemagglutinin [23,24,25,26].

## 3. Results and Discussion

### 3.1. Evaluated Affinities of Measles Virus H Protein (MVH) to Signaling Lymphocyte-Activation Molecule (SLAM), Nectin-4, and CD46 by the Fragment Molecular Orbital (FMO) Method

The IFIE sums of the residues between MVH and each chain of receptors obtained in the present FMO calculations are shown in Table 1. The calculated values of IFIE sums between MVH and the respective three receptors are substantially different. The SLAM molecule (Chain B in PDB code 3ALZ) showed the strongest attractive interaction with MVH among the three receptors. Among the various types of binding interactions, the contribution of electrostatic forces for repulsive and attractive interactions is predominant. Table 1 shows that only the SLAM receptor has a total positive charge (+2) that is favorable for an attractive interaction with the negatively charged MVH, thus leading to the largest negative value of the IFIE sum. Weaker binding interactions of Nectin-4 and CD46 receptors with MVH are due to their electric charges being negative (-5 and -4, respectively).

To show the contribution of each amino acid residue to the affinity, Table 2 lists the top 20 amino acid residues in MVH that show strongly attractive interactions with the respective three receptors, where only the IFIEs of fragments located within 5 Å from each residue are summed to obtain the IFIE sums. Table 2 also lists the amino acid pairs between MVH and the three receptors that show the largest attractive IFIE for each high-rank residue in MVH, indicating the most important partner residue (designated as “receptor a.a. with the highest (1st) affinity” in Table 2) in the receptors; their contribution to the IFIE sum associated with each residue in MVH is also indicated as “IFIE-1st” along with its ratio to the respective IFIE sum.

Thus, Table 2 demonstrates the following results: First, charged amino acid residues (marked by + or − in Table 2) forming an ion pair or salt bridge have higher IFIEs. Second, six, one and three ion pairs are found in the MVH–SLAM complex, the MVH–Nectin-4 complex, and the MVH–CD46 complex, respectively, in the list representing residue pairs that are essential for molecular recognition. Third, there are a number of hydrophobic residues (marked by asterisk in Table 2) in MVH that play vital roles in the interactions with the receptors.

In Table S1 of Supplementary Material, we also list the calculated IFIE results for the second and third most important residues in the three receptors (designated as “2nd receptor a.a.” and “3rd receptor a.a.”, respectively) that show attractive interactions with the respective MVH residues shown in Table 2. We thus see that several uncharged (neutral) residues in the receptors play important roles in the intermolecular interactions with MVH (see also Table 3). For example, Pro102 in Nectin-4 shows attractive interactions with Tyr543 and Pro458 in MVH as the 3rd and 2nd important residues, respectively, thus contributing to the binding.

### 3.2. Distribution of Amino Acid Residues on MVH Showing Significant Interactions with the Three Receptors

**Figure 2:**
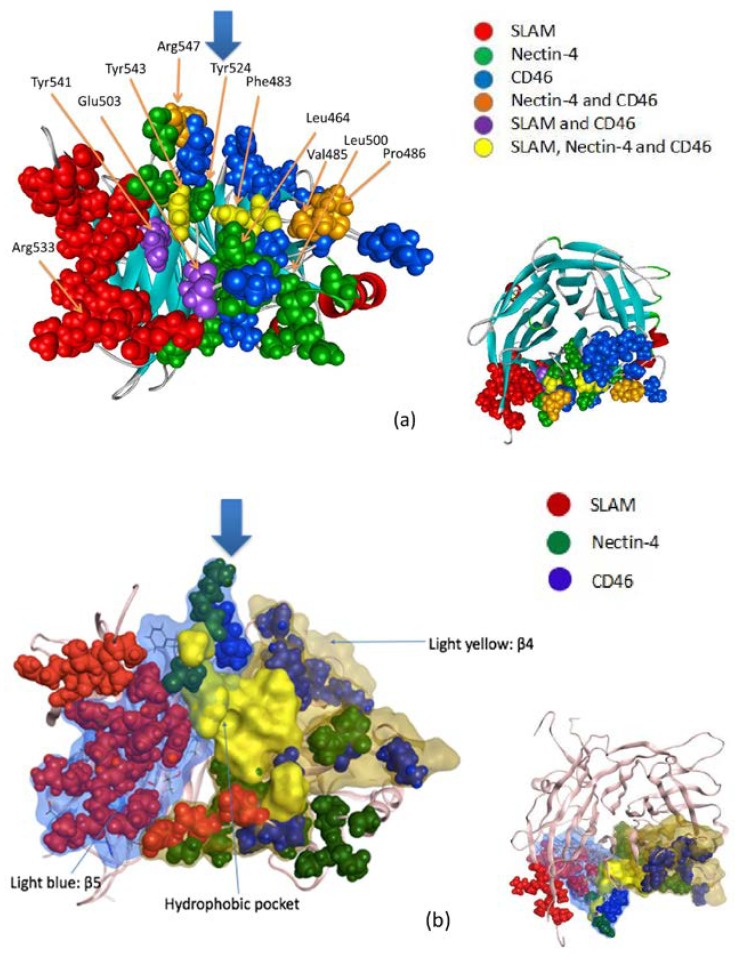
Amino acid residues on the interface of MVH binding to the three receptors. (**a**) Colored spheres represent the residues of MVH that show significantly attractive interactions with the three receptors; red, green, and blue spheres indicate the amino acid residues with high affinity to SLAM, Nectin-4, and CD46, respectively; orange indicates high affinity to both Nectin-4 and CD46; violet indicates high affinity to both SLAM and CD46; yellow indicates high affinity to all of SLAM, Nectin-4, and CD46. Some important residues are explicitly indicated. The smaller figure at the lower right shows the top view from the direction indicated by the thick arrow in the main figure. (**b**) Corresponding to (**a**), the hydrophobic pocket in MVH is represented in yellow from the same direction as (**a**). In addition, the β4 and β5 strand regions are depicted in light yellow and light blue, respectively. The smaller figure at the lower right shows the top view from the direction indicated by the thick arrow in the main figure.

**Figure 3:**
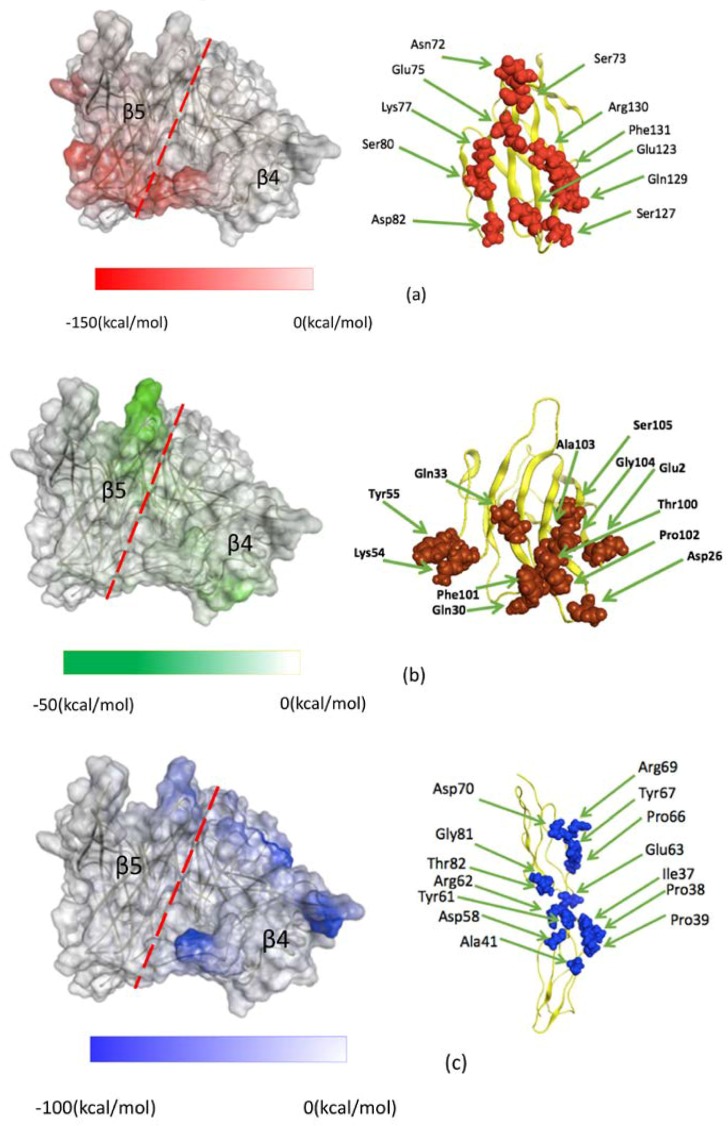
Interfaces of MVH and the respective three receptors. (**Left**) β4 and β5 strand regions on the MVH surface that show strongly attractive interactions with the three receptors: (**a**) SLAM (red), (**b**) Nectin-4 (green), and (**c**) CD46 (blue). The deepness of the color represents the strength of the IFIE sum associated with the fragment (MVH residue) at that position. The dashed line represents the approximate boundary between the β4 and β5 regions. (**Right**) Residues on the receptor surface that show importantly attractive interactions with MVH: (**a**) SLAM, (**b**) Nectin-4, and (**c**) CD46.

Using the results of FMO-IFIE analysis shown in Table 2, Figure 2 visually illustrates the residues of the interface in MVH that are important for binding to the receptors. In Figure 2a, the residues in MVH that have strongly attractive interactions with the respective three receptors are shown in different colors, where some common residues for two or three receptors are also shown. For example, Phe483 and Tyr543 (represented in yellow in Figure 2a) seem to be important for binding to the three receptors in common (see also Table 3 below). Several important amino acid residues are observed around the hydrophobic pocket in the β4 (residues 448–507) and β5 (residues 524–556) strand regions (see Figure 2b). In addition, Figure 3 (left-hand side) depicts the surface regions of MVH that are responsible for binding to the three receptors, where the β4 and β5 strand regions are illustrated with the approximate boundary (shown by a dashed line); the strength of the (attractive) IFIE sums associated with the pertinent residues in MVH is indicated by the intensity of the color. Figure 3 (right-hand side) also illustrates the amino acid residues of the three receptors which showed attractive IFIEs with MVH in the present FMO calculations.

Table 3 lists the IFIE sums between the three receptors and some important residues in MVH, i.e., (1) those in the hydrophobic region at the MVH-receptor interface and (2) those with the strongest (top five) attractive interactions with SLAM. These residues were selected to study the relative importance of hydrophobic and electrostatic interactions in MVH-receptor binding. As shown in Table 3, Phe483 and Tyr543 in the hydrophobic pocket have attractive interactions with all three receptors whose IFIE amplitudes are higher than 5 kcal/mol. Leu464 and Leu500 show significantly attractive interactions with Nectin-4 (−10.9 and −11.7 kcal/mol, respectively). Contributions of the hydrophobic pocket to the interactions between MVH and the respective receptors, SLAM, Nectin-4 and CD46, were calculated to be 4.2%, 17.4% and 3.1%, respectively. This indicates that the contribution of the hydrophobic pocket is the highest with Nectin-4. This result is consistent with Figure 2 and Figure 3, which depict that the binding interface of Nectin-4 is overlapped with the hydrophobic pocket of MVH more substantially than in the other two receptors. On the other hand, the contribution of the top 5 ionic bridges in the interaction between MVH and SLAM was 65.4%, which was much higher than that of the hydrophobic pocket (Table 3). In contrast, only Arg547 of MVH was contained in the top 5 strongly interacting residues as a charged residue in Nectin-4 binding (Table 2), whose contribution, −46.4 kcal/mol, was smaller in magnitude than the sum of the hydrophobic residues mentioned above, −58.8 kcal/mol (Table 3), again indicating the relative importance of hydrophobic interaction in the MVH–Nectin-4 complex.

### 3.3. Comparison of Calculated MVH-Receptor Affinities with Those of Experimental Observations

In this subsection, we compare the calculated results above with the experimental suggestions based on the molecular structures of MVH-receptor complexes. X-ray crystallography experiments have revealed that three receptors, SLAM, CD46, and Nectin-4, bind to adjacent surface areas of the hydrophobic pocket of MVH. Earlier crystallographic studies [11,12,13] pointed out some key amino acid residues of MVH that would be essential for binding to the respective receptors. These include Leu464, Leu482, Phe483, Leu500, Tyr524, Tyr541, Tyr543, and Ser548 around the hydrophobic pocket in the β4 and β5 strand regions (see Figure 2 and Figure 3). Furthermore, charged residues such as Asp, Glu, Arg, and Lys are also supposed to play a vital role in binding owing to electrostatic interactions.

A number of these residues in MVH can be specified as partners showing strongly attractive IFIEs with their receptor residues in the FMO calculations. Figure 2 and Figure 3 and Table 2 and Table 3 illustrate that the residues in the hydrophobic pocket provide a common platform for attractive interactions between the three receptors and MVH. On the other hand, the charged residues in MVH showed high IFIE values with the respective three receptors in a specific manner. The present FMO analysis thus indicates overall agreement between (crystallographic) experimental and computational results. (For the comparison with virological experiments, see the following sections.)

Concerning the important residues in the receptors, for the interactions between MVH and SLAM (Table 2), we observed that Lys77 and Glu123 in SLAM (see Figure 3a) play pivotal roles in binding to MVH in the FMO analysis. These top two amino-acid residues of human SLAM regarding the strength of attractive interaction are known to be well conserved among the reported mammalian SLAMs [27], thus indicating that they are actually important in the interaction between SLAM and MVH, and suggesting that they could be essential for morbillivirus binding to the host receptors.

Concerning the residues of MVH around the hydrophobic pocket in the β4 and β5 strand regions, in case of the Nectin-4 receptor, five non-charged residues, Leu464, Phe483, Leu500, Tyr543, and Ser548, suggested as important in the molecular recognition structurally [13], were among the top 10 strongly attractive amino-acid residues of MVH interacting with Nectin-4 (Table 2). Thus, the present FMO calculations were consistent with the results of the X-ray experimental data [13], indicating that this calculation provides a useful tool for analyzing and evaluating the interactions between MVH and its receptors. The data also showed that the calculation in vacuo is good enough for estimating or ranking the binding energy, although an improved approach incorporating the solvent effect is desirable in future studies. In addition, this approach gives basic quantitative information for explaining the experimental results of binding affinity based on structural data.

### 3.4. Effects of Amino Acid Substitutions in MVH on the Binding Affinity

We then substituted several amino acid residues in MVH and studied the effects of these substitutions on the IFIE sums between MVH and the receptors. We selected mutations in MVH for which those residues in the wild-type MVH showed strongly attractive interactions with the receptors or those which have been reported as important by mutational experiments [18,28,29,30,31]. Table 4 summarizes the calculated results in which the changes in IFIE sums due to the mutations are shown in comparison with the reported experimental (virological) results for binding affinity.

**Figure 4:**
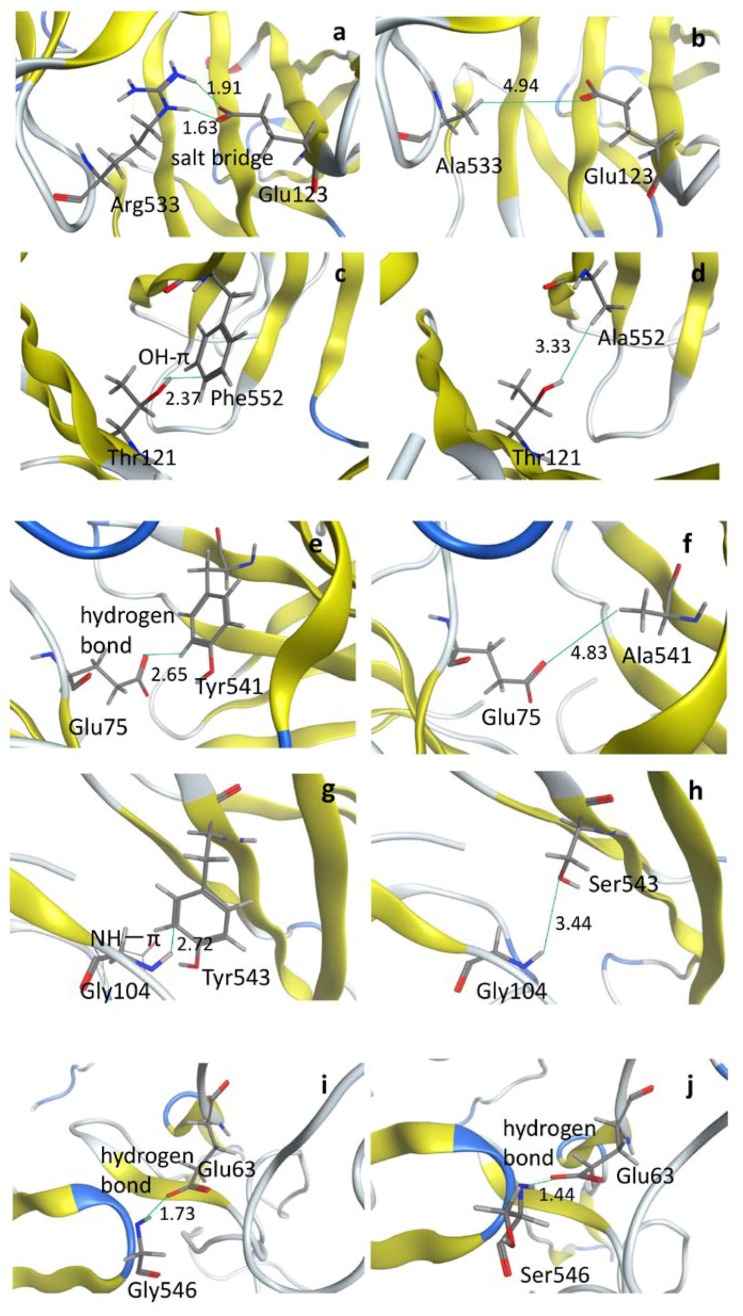
Local binding structures (**a**) before and (**b**) after the R533A mutation in the MVH–SLAM complex; (**c**) before and (**d**) after the F552A mutation in the MVH–SLAM complex; (**e**) before and (**f**) after the Y541A mutation in the MVH–SLAM complex; (**g**) before and (**h**) after the Y543S mutation in the MVH–Nectin-4 complex; and (**i**) before and (**j**) after the G546S mutation in the MVH–CD46 complex. The residues important for binding are depicted in the stick representation, where grey, red, blue, and white colors refer to carbon, oxygen, nitrogen, and hydrogen atoms, respectively. Numerals indicate the respective distances (in units of Å) between two atoms forming important chemical bonds depicted by green lines.

The present FMO calculation showed that the binding pair interaction between Arg533 of MVH and Glu123 of SLAM was the highest [−122 kcal/mol (=17.2% of total IFIE)] among the examined residue pairs (Table 2). When the mutation of R533A in MVH was performed in the computer simulation for the MVH–SLAM complex, the magnitude of the attractive IFIE sum was reduced by 71.0 kcal/mol to −636.7 kcal/mol (Table 4). This result of the FMO calculation is consistent with the virological result that the substitutional mutant R533A of the MVH–SLAM complex actually lost binding activity [28,29]. The Arg533 of MVH should be essential for MV infection owing to its high binding (pair interaction) energy with Glu123 of SLAM. Figure 4a,b illustrate the structural change before and after the mutation in which the salt bridge between Arg533 and Glu123 was lost because of the R533A mutation. While the electrostatic interactions were somewhat overestimated in this FMO calculation because the present calculation was conducted in vacuo, the solvent effect was not considered to be so serious as to complicate the detection of important pair interactions because of the importance of direct interaction between the residues [17,23,24,25,26]. Although the calculated FMO-binding energy (IFIE sum) for the mutant is still about 90% of that in the wild type, it is not the ratio (10% reduction) but the substantial reduction in the strength of attractive IFIE (71.0 kcal/mol) that is essential for realizing the observed loss of binding (Table 4), because the overestimation of the magnitude of electrostatic interactions in the FMO calculations in vacuo makes the evaluated binding energy too attractive. Empirically, the reduction in attractive IFIE by about 50 kcal/mol and 10 kcal/mol for charged and neutral cases, respectively, could explain the loss of binding affinity due to mutation (see also the cases below).

In addition, the significant reduction (by 60.9 kcal/mol) in the attractive IFIE for the D530A mutation (Table 4) accounts for the loss of binding activity in the corresponding virological experiment [28,29]. Moreover, virological experiments [28,29] have shown the reduction of binding activity in F552A, P554A, and D507A mutants for the MVH–SLAM complex, which agree with the corresponding FMO results in Table 4. For example, in the case of the F552A mutant in the MVH–SLAM complex, the attractive IFIE sum was reduced by 26.8 kcal/mol. The reason for this reduction of binding in F552A was the loss of OH-π interaction between Phe552 and Thr121 due to the mutation (Figure 4c,d). Furthermore, noting that Tyr541, a hydrophobic residue in MVH, showed a relatively strong attractive interaction with SLAM (Table 2), we made the computational mutation of Y541A, which showed reduction of the attractive IFIE sum by 13.2 kcal/mol (Table 4). Concerning this change in attractive IFIE sum for Y541A, the associated structural change illustrated in Figure 4e,f indicates that loss of the attractive (hydrogen bond-like) interaction between Tyr541 in MVH and Glu75 in SLAM is a dominant cause. Because the virological experiment for this mutation has not yet been performed, it would be interesting to assess it experimentally. Altogether, the present calculated data for the substitutional mutant MVHs regarding binding to SLAM agreed well with the virological experimental data (Table 4). This indicates that the research strategy using the FMO method is dependable for analyzing the interaction of viral H with its receptors. This method would thus be applicable for predicting such vital interactions in an exhaustive manner before performing the virological experiments.

Regarding the complex of MVH and Nectin-4, the present FMO calculation showed that the substitution Y543S caused a reduction of attractive IFIE by 6.5 kcal/mol compared to that in the wild type (Table 4). This mutation has been experimentally reported to result in the loss of binding activity [4,30]. Figure 4g,h illustrate the structural change before and after the Y543S mutation, thus showing the loss of NH-π interaction between Tyr543 and Gly104. Since this NH-π interaction is of the dipole-induced dispersion type, the reduction of attractive IFIE by 6.5 kcal/mol, which is smaller than in the (overestimated) charged residue case, could cause the loss of the binding between MVH and Nectin-4. In this context, Tyr543 may not be so important for the MVH binding to SLAM [30] due to the difference in associated IFIE-sum between Y543-SLAM (−5.1 kcal/mol) and Y543-Nectin-4 (−16.8 kcal/mol), as shown in Table 3. It is also noted that the charged residues play more important roles in the case of binding with SLAM (see above).

Among the high binding-affinity residues of MVH for CD46 (Table 2), the present FMO calculation showed that the IFIE sums remarkably changed toward more repulsive interactions in the mutants of Y481N and Y481A (Table 4). These data concurred with the experimental data that the mutants Y481N and Y481A actually lose their binding activity to CD46 [18,28,31]. In this case, the loss of hydrogen bonding between Tyr481 and Pro66 was found to be the predominant cause for reduction in binding.

In contrast, the FMO calculation showed an increase in attractive interaction for G546S (Table 4), which did not agree with the experimental results indicating the loss of binding between MVH and CD46 [4,30,31]. The local interactions between Glu63 of CD46 and Gly546 (or mutated Ser546) of MVH are depicted in Figure 4i,j. The changes in IFIEs between some important residues of CD46 and those of MVH are shown in Table S2 of Supplementary Material. We thus see that the increase in the attractive IFIE sum by 23.8 kcal/mol (Table 4) was mainly caused by the increase in the attractive IFIE between Glu63 and Arg547 (by 17.6 kcal/mol), whose distance was decreased (by 0.3 Å) because of the G546S mutation. Such an “indirect” effect of mutation accompanying structural changes in unmutated residues was also observed in the case of influenza virus hemagglutinin [26]. Concerning this exceptional case, the simulation methods may need improvements including how to prepare the complex structures more accurately, because the FMO-IFIE values, especially the electrostatic ones, are very sensitive to the molecular structures employed for the calculations. Relevant inclusion of solvent effects would also be necessary. These issues remain to be elucidated in the future study.

Finally, when an *in silico* mutant with five simultaneous substitutions of E471A, K477A, K488A, E503A and R547A was made, the (attractive) IFIE sum between MVH and CD46 was significantly reduced from −285.7 kcal/mol to −142.3 kcal/mol, to about a half of that for the wild type, showing an example of the cumulative effect of multiple amino acid substitutions (Table 4). Considering the substantial reduction in attractive IFIE by more than 100 kcal/mol, we may explore the promising possibility of utilizing these cumulative effects for therapeutic applications in the future (see below).

Taken together, the present FMO-IFIE analysis agreed well with the reported experimental results concerning the molecular recognition between MVH and the three receptors in the cases of both wild-type and mutant hemagglutinin (H) proteins.

### 3.5. Designs for SLAM-Blind MVH and MVH-Binding Inhibitor

Natural MV infection has been reported to have anti-tumor activity [32,33]. This has led to the development of live attenuated MV-based therapy for some cancers in which CD46 is highly expressed [34,35]. The therapy depends on the targeting specificity and systemic infection of MV. MV-modified oncolytic virotherapy is currently being tested in clinical trials against ovarian cancer and multiple myeloma [36]. The FMO approach may then provide valuable information for analyzing the designed MVH.

SLAM is the principal receptor when wild-type MV infects the host. Recombinant MV which is selectively blind to SLAM is reported to alter the target specificity of MV and affect cancer cells [18,19]. Recently Nectin-4 has been reported to be up-regulated in a variety of cancer cells [37,38]. SLAM-blind MV with only R533A substitution has shown oncologic efficacy on lung and pancreatic cancer cells implanted in severe combined immune deficiency (SCID) mice [39,40,41]. Table 3 above shows that five amino acid residues in MVH, Asp507, Asp505, Arg533, Arg556, and Asp530, play pivotal roles in binding to SLAM. Because these residues do not show significant attractive interactions with other receptors (Nectin-4 and CD46), their substitution with other residues would be effective for producing measles viruses that are specifically unable to bind SLAM, without change in binding to other receptors. Mutations at Arg533 and/or Asp530 in MVH such as R533A and/or D530A (Table 4) actually showed a reduction in their binding energies with SLAM. Thus, as the computational approach using the FMO method can predict the effect of amino acid substitution on binding with each of the three receptors, it can contribute to generating a newly designed, modified measles virus with higher specificity to the target cells and higher safety for oncolytic virotherapy.

Computational results based on the FMO method could also provide useful information for designing effective inhibitors against measles viruses. While measles can be controlled and eliminated with increased vaccine coverage, developments of novel drugs may still be helpful for the treatment of measles patients in developing countries, and also of sporadically infected patients in advanced countries. There are target regions and several specific residues around the interfaces between MVH and the three receptors, as elucidated in the calculations above. Concerning the design for MVH-binding inhibitors, Table 3 shows that Phe483 has attractive interactions with all three receptors whose (attractive) IFIE amplitudes are higher than 6 kcal/mol. This residue can, therefore, be regarded as a potential target for effective inhibitors against measles viruses. On the other hand, if residues in MVH that specifically interact with one of the receptors are targeted, we could design inhibitors which only suppress the binding to that receptor.

## 4. Conclusions

In this study, we applied the FMO method to exhaustively analyze intermolecular interactions between MVH and its three receptors, SLAM, Nectin-4, and CD46. The interactions between amino acid residues were evaluated in terms of IFIEs and their sums, which provided quantitative verifications of important residues inferred from X-ray crystallography data on the MVH-receptor complex structures. In the present analysis, we found a feature that the non-polar residues in the interfacial hydrophobic pocket of MVH are used for binding to the three receptors in common, whereas the charged residues are employed for specific molecular recognition of different receptors. The advantage of FMO computational analysis is that it is possible to comprehensively identify those residues important for binding or molecular recognition without experimentally performing the alanine scanning studies. In addition, computational mutations in MVH were actually performed for several amino-acid residues, which again showed reasonable consistency between the FMO calculations and virological experiments. Through these computational analyses, we expect that the FMO-based approach could provide a powerful tool for designing novel inhibitors against MV infection and engineered MVs for cancer therapy, while there remain a number of elements to be considered in future study such as use of accurate molecular structure, solvent effect, cellular environment and other biological conditions to attain more satisfactory agreement with virological experiments.

## Figures and Tables

**Table 1:** Total IFIEs (IFIE sums) in complexes between MVH and three types of receptors, SLAM, Nectin-4, and CD46.

Receptor	SLAM	Nectin-4	CD46
Protein Data Bank (PDB) code	3ALZ	4GJT	3INB
Chain designation in PDB	Chain B ^(3)^	Chain B ^(4)^	Chain D ^(5)^
Receptor charge ^(1)^	+2	−5	−4
MVH charge ^(2)^	−1	−2	−2
Total IFIE (kcal/mol)	−707.7	−173.8	−285.7

^(1)^ Electric charges of receptor chains in the complexes with MVH employed in the FMO calculations; ^(2)^ Electric charges of MVH are different according to the amino-acid sequences of employed PDB structures; ^(3)^ Chain B of SLAM receptor complexed with MVH monomer (Chain A) in PDB structure of 3ALZ; ^(4)^ Chain B of Nectin-4 receptor complexed with MVH monomer (Chain A) in PDB structure of 4GJT; ^(5)^ Chain D of CD46 receptor complexed with Chain A of MVH dimer in PDB structure of 3INB.

**Table 2:** Ranking of MVH residues showing strongly attractive interactions with the three receptors. (**a**) SLAM; (**b**) Nectin-4; (**c**) CD46.

Rank	(a) SLAM (Chain B in PDB Code 3ALZ)				
	MVH Amino Acid (a.a.) Position ^(1)^	IFIE Sum ^(2)^ with the Receptor	Receptor a.a. with the Highest (1st) Affinity ^(3)^	IFIE-1st ^(4)^ with the Receptor a.a.	IFIE-1st/IFIE Sum Ratio ^(5)^
		(kcal/mol)		(kcal/mol)	
1	Asp507(−)	−133.2	Lys77(+)	−80.0	60%
2	Asp505(−)	−107.7	Lys77(+)	−106.3	99%
3	Arg533(+)	−80.5	Glu123(−)	−122.0	152%
4	Arg556(+)	−74.2	Glu123(−)	−45.7	62%
5	Asp530(−)	−67.6	Lys77(+)	−66.4	98%
6	Glu503(−)	−53.4	Lys77(+)	−48.0	90%
7	Arg195(+)	−36.7	Ser127	−24.9	68%
8	Thr193(*)	−32.6	Arg130(+)	−15.3	47%
9	Phe552(*)	−18.6	Glu 75(−)	−9.2	49%
10	Tyr551(*)	−18.0	Arg130(+)	−17.8	99%
11	Tyr553(*)	−14.1	Ser 127	−7.2	51%
12	Ser532(*)	−13.1	Ser 80	−4.8	37%
13	Phe483(*)	−12.3	Asn72	−11.8	96%
14	Gly196(*)	−9.3	Ser 127	−11.7	126%
15	Pro554	−8.9	Glu123(−)	−9.9	111%
16	Tyr541(*)	−8.6	Glu 75(−)	−6.3	73%
17	Thr192(*)	−7.5	Phe 131	−14.2	189%
18	Ile194(*)	−7.3	Gln129	−8.5	116%
19	Val534(*)	−6.0	Asp82(−)	−4.7	78%
20	Tyr543(*)	−5.1	Ser73	−3.2	63%
**Rank**	**(b) Nectin-4 (Chain B in PDB Code 4JGT)**				
1	Arg547(+)	−46.4	Glu2(−)	−42.1	91%
2	Ser550(*)	−29.5	Asp26(−)	−25.8	87%
3	Gln391	−27.5	Tyr55	−24.4	89%
4	Tyr543(*)	−16.8	Gly104	−5.8	35%
5	Thr392(*)	−16.8	Lys 54(+)	−18.3	109%
6	Leu500(*)	−11.7	Lys 54(+)	−6.3	54%
7	Leu464(*)	−10.9	Thr100	−6.1	56%
8	Phe483(*)	−10.0	Ser105	−5.3	53%
9	Gly506(*)	−9.3	Gln 30	−9.3	100%
10	Ser548(*)	−9.1	Glu2(−)	−10.2	112%
11	Gly465(*)	−7.1	Ala 103	−11.7	165%
12	Gly388(*)	−6.3	Tyr 55	−4.6	73%
13	Tyr499(*)	−5.9	Lys 54(+)	−5.6	95%
14	Tyr524(*)	−3.9	Gly 104	−2.9	74%
15	Lys460(+)	−3.1	Phe101	−3.1	100%
16	Val485(*)	−2.3	Gln33	−1.6	70%
17	Pro486	−2.1	Gln 33	−1.7	81%
18	Ala463(*)	−1.9	Phe 101	−1.9	100%
19	Pro458	−1.9	Ala103	−2.0	105%
20	Ile390(*)	−1.1	Lys 54(+)	−1.1	100%
**Rank**	**(c) CD46 (Chain D in PDB Code 3INB)**				
1	Lys477(+)	−66.2	Asp70(−)	−67.4	102%
2	Glu503(−)	−36.1	Ala41	−23.3	65%
3	Gly546(*)	−33.7	Glu63(−)	−27.2	81%
4	Lys488(+)	−29.6	Gly81	−29.9	101%
5	Tyr481(*)	−22.1	Pro66	−14.5	66%
6	Glu471(−)	−17.9	Arg69(+)	−51.4	287%
7	His448	−14.2	Arg69(+)	−10.0	70%
8	Pro501	−7.3	Asp58(−)	−5.6	77%
9	Phe483(*)	−6.6	Tyr 61	−3.7	56%
10	Pro486	−6.4	Thr 82	−4.0	63%
11	Tyr543(*)	−6.3	Ile37	−15.5	246%
12	Arg547(+)	−6.3	Glu63(−)	−45.4	721%
13	Thr498(*)	−6.2	Tyr 61	−6.2	100%
14	Val485(*)	−4.3	Arg62(+)	−4.5	105%
15	Tyr541(*)	−4.3	Pro38	−1.7	40%
16	His495	−3.6	Thr 82	−4.2	117%
17	Val451(*)	−3.4	Tyr 67	−1.8	53%
18	Pro545	−3.3	Glu63(−)	−1.9	58%
19	Thr469(*)	−2.2	Tyr 67	−2.2	100%
20	Leu462(*)	−2.1	Pro39	−1.5	71%

^(1)^ “MVH amino acid (a.a.) position” shows amino acid residues of MVH and their sequence number with electric charge (+ or −) or hydrophobicity (*) in the parentheses. Ranking of the MVH a.a. is descending from the highest (most attractive) IFIE value on the top to the lower IFIE values downward; ^(2)^ “IFIE sum” is the summation of the IFIE values with the residues of the receptor located within the pair distance of 5 Å; ^(3)^ “Receptor a.a. with the highest (1st) affinity” indicates the most attractive amino-acid residue of the receptor to the corresponding MVH residue on the same line. Its position is shown as the sequence number with its electric charge (+ or −) in the parentheses; ^(4)^ “IFIE-1st” is the IFIE between the designated MVH a.a. and the receptor a.a. with the highest (1st) affinity; ^(5)^ Contribution of the receptor a.a. with the highest (1st) affinity to the IFIE sum of the single MVH residue and the receptor residues was evaluated as a ratio between the IFIE-1st and the IFIE sum.

**Table 3:** Characteristic IFIEs between amino acid residues of MVH and three receptors, SLAM, Nectin-4, and CD46. The summations of these IFIE values over the MVH residues and their contributions to the total IFIE values in Table 1 are also shown.

Hydrophobicity	Amino Acid Residues of MVH	IFIE Sum (kcal/mol) with Receptor Residues within 5 Å ^(a)^		
		SLAM (Chain B in PDB Code 3ALZ)	Nectin-4 (Chain B in PDB Code 4JGT)	CD46 (Chain D in PDB Code 3INB)
Hydrophobic (neutral) amino acids	Leu464 ^(b)^	−0.7	−10.9	−0.4
	Leu482 ^(b)^	−1.4	−0.2	2.5
	Phe483 ^(b)^	−12.3	−10.0	−6.6
	Leu500 ^(b)^	0.2	−11.7	9.3
	Tyr524 ^(b)^	−0.6	−3.9	−1.7
	Tyr541 ^(b)^	−8.6	3.7	−4.3
	Tyr543 ^(b)^	−5.1	−16.8	−6.3
	Ser548 ^(b)^	−1.2	−9.1	−1.3
	IFIE sum of the above values	−29.7	−58.8	−8.8
	Contribution to the MVH-receptor interaction ^(c)^	4.2%	17.4%	3.1%
Hydrophilic (charged) amino acids	Asp507 ^(d)^	−133.2	— ^(^^f)^	—
	Asp505 ^(d)^	−107.7	−0.4	—
	Arg533 ^(d)^	−80.5	—	—
	Arg556 ^(d)^	−74.2	—	—
	Asp530 ^(d)^	−67.6	—	—
	IFIE sum above	−463.0		
	IFIE sum/Total IFIE ^(e)^	65.4%		

^(a)^ IFIEs were calculated between the MVH residue and the residues of receptors within the range of 5 Å from the MVH residue; ^(b)^ Amino acid residues in the hydrophobic pocket of MVH; ^(c)^ Contribution of the residues in the hydrophobic pocket to the MVH-receptor interaction calculated with the partial IFIE sum divided by the total IFIE in Table 1; ^(d)^ Top 5 amino acid residues of MVH calculated to have the most attractive IFIEs with SLAM; ^(e)^ IFIE sum divided by the total IFIE in Table 1; ^(f)^ “—” indicates that there is no residue in the receptor within the distance of 5 Å from the respective MVH residue.

**Table 4:** Effects of mutations of MVH residues on the binding affinities (IFIE sums in units of kcal/mol) with the three receptors.

Receptor	MVH Mutation	Strand Region	IFIE (before Mutation)	IFIE (after Mutation)	ΔIFIE * (kcal/mol)	Major Cause for IFIE Change	Virological Experimental Results
SLAM	R533A	β5	−707.7	−636.7	71.0	Loss of salt bridge	+ + [28]
	D505A	β4–β5	−707.7	−608.7	98.9	Loss of salt bridge	n.r. ^$^
	D507A	β4–β5	−707.7	−576.8	130.9	Loss of salt bridge	+ [29]
	D530A	β5	−707.7	−646.8	60.9	Loss of salt bridge	+ + [29]
	E503A	β4–β5	−707.7	−661.6	46.0	Loss of salt bridge	n.r.
	P554A	β5–β6	−707.7	−675.4	32.2	Loss of van der Waals interaction	+ [28]
	F552A	β5–β6	−707.7	−680.9	26.8	Loss of OH-π	+ [28]
	Y541A	β5	−707.7	−694.5	13.2	Loss of hydrogen bond	n.r.
	L482R	β4	−707.7	−713.8	−6.1	Increase of electrostatic interaction	n.r.
Nectin-4	Y543S	β5	−173.8	−167.3	6.5	Loss of NH-π	+ + [30]
CD46	G546S	β5	−285.7	−309.6	−23.8	Increase of electrostatic interaction	+ + [31]
	Y481A	β5	−285.7	−265.5	20.3	Loss of hydrogen bond	+ + [18]
	Y481N	β5	−285.7	−267.8	17.9	Loss of hydrogen bond	+ + [28]
	E471A ^#^	β4	−285.7	−142.3 ^#^	143.5 ^#^	Loss of van der Waals interaction	n.r.
	K477A ^#^	β4					
	K488A ^#^	β4					
	E503A ^#^	β4–β5					
	R547A ^#^	β5					

Following the second column for mutation specification, the associated secondary structure of MVH, the change in IFIE value (see * below), major cause for IFIE change, and binding change result by virological experiment (+, + + with references or n.r.; see below) are shown. * IFIE(after mutation)—IFIE(before mutation). + Partial loss of the binding between the mutated MVH and the receptor has been reported in the literature shown in the parentheses. + + Loss of the binding of the mutated MVH to the receptor has been reported in the literature shown in the parentheses. ^$^ n.r.: not reported. ^#^ Computational mutant with five multiple substitutions.

## Supplementary Materials

The following materials are available online at http://www.mdpi.com/1999-4915/10/5/236/s1, Table S1 and Table S2.

## References

[1] Bulter D. (2015). Measles by the numbers: A race to eradication. Nature.

[2] Tatsuo H., Ono N., Tanaka K., Yanagi Y. (2000). SLAM (CDw150) is a cellular receptor for measles virus. Nature.

[3] Schwartzberg P.L., Mueller K.L., Qi H., Cannons J.L. (2009). SLAM receptors and SAP influence lymphocyte interactions, development and function. Nat. Rev. Immunol..

[4] Tahara M., Takeda M., Shirogane Y., Hashiguchi T., Ohno S., Yanagi Y. (2008). Measles virus infects both polarized epithelial and immune cells by using distinctive receptor-binding sites on its hemagglutinin. J. Virol..

[5] Noyce R.S., Bondre D.G., Ha M.N., Lin L.T., Sisson G., Tsao M.S., Richardson C.D. (2011). Tumor cell marker PVRL4 (nectin 4) is an epithelial cell receptor for measles virus. PLoS Pathog..

[6] Muhlebach M.D., Mateo M., Sinn P.L., Prufer S., Uhlig K.M., Leonard V.H., Navaratnarajah C.K., Frenzke M., Wong X.X., Sawatsky B. (2011). Adherens junction protein nectin-4 is the epithelial receptor for measles virus. Nature.

[7] Naniche D., Wild T.F., Rabourdin-Combe C., Gerlier D. (1992). A monoclonal antibody recognizes a human cell surface glycoprotein involved in measles virus binding. J. Gen. Virol..

[8] Dorig R.E., Marcil A., Chopra A., Richardson C.D. (1993). The human CD46 molecule is a receptor for measles virus (Edmonston strain). Cell.

[9] Lin L.-T., Richardson C.D. (2016). The host cell receptors for measles virus and their interaction with the viral hemagglutinin (H) protein. Viruses.

[10] Hashiguchi T., Kajikawa M., Maita N., Takeda M., Kuroki K., Sasaki K., Kohda D., Yanagi Y., Maenaka K. (2007). Crystal structure of measles virus hemagglutinin provides insight into effective vaccines. Proc. Natl. Acad. Sci. USA.

[11] Santiago C., Celma M.L., Stehle T., Casasnovas J.M. (2010). Structure of the measles virus hemagglutinin bound to the CD46 receptor. Nat. Struct. Mol. Biol..

[12] Hashiguchi T., Ose T., Kubota M., Maita N., Kamishikiryo J., Maenaka K., Yanagi Y. (2011). Structure of the measles virus hemagglutinin bound to its cellular receptor SLAM. Nat. Struct. Mol. Biol..

[13] Zhang X., Lu G., Qi J., Li Y., He Y., Xu X., Shi J., Zhang C.W., Yan J., Gao G.F. (2013). Structure of measles virus hemagglutinin bound to its epithelial receptor nectin-4. Nat. Struct. Mol. Biol..

[14] Suzuki Y. (2017). Predicting receptor functionality of signaling lymphocyte activation molecule for measles virus hemagglutinin by docking simulation. Microbiol. Immunol..

[15] Kitaura K., Ikeo E., Asada T., Nakano T., Uebayasi M. (1999). Fragment molecular orbital method: An approximate computational method for large molecules. Chem. Phys. Lett..

[16] Nakano T., Kaminuma T., Sato T., Akiyama Y., Uebayasi M., Kitaura K. (2000). Fragment molecular orbital method: Application to polypeptides. Chem. Phys. Lett..

[17] Tanaka S., Mochizuki Y., Komeiji Y., Okiyama Y., Fukuzawa K. (2014). Electron-correlated fragment-molecular-orbital calculations for biomolecular and nano systems. Phys. Chem. Chem. Phys..

[18] Nakamura T., Peng K.W., Vongpunsawad S., Harvey M., Mizuguchi H., Hayakawa T., Cattaneo R., Russell S.J. (2004). Antibody-targeted cell fusion. Nat. Biotechnol..

[19] Nakamura T. (2009). Recombinant measles virus for cancer therapy. Drug Deliv. Syst..

[20] Msaouel P., Iankov I.D., Dispenzieri A., Galanis E. (2012). Attenuated oncolytic measles virus strains as cancer therapeutics. Curr. Pharm. Biotechnol..

[21] Aref S., Bailey K., Fielding A. (2016). Measles to the rescue: A review of oncolytic measles virus. Viruses.

[22] (2016). Molecular Operating Environment (MOE) ver. 2016.08.

[23] Iwata T., Fukuzawa K., Nakajima K., Aida-Hyugaji S., Mochizuki Y., Watanabe H., Tanaka S. (2008). Theoretical analysis of binding specificity of influenza viral hemagglutinin to avian and human receptors based on the fragment molecular orbital method. Comput. Biol. Chem..

[24] Takematsu K., Fukuzawa K., Omagari K., Nakajima S., Nakajima K., Mochizuki Y., Nakano T., Watanabe H., Tanaka S. (2009). Possibility of mutation prediction of influenza hemagglutinin by combination of hemadsorption experiment and quantum chemical calculation for antibody binding. J. Phys. Chem. B.

[25] Yoshioka A., Fukuzawa K., Mochizuki Y., Yamashita K., Nakano T., Okiyama Y., Nobusawa E., Nakajima K., Tanaka S. (2011). Prediction of probable mutations in influenza virus hemagglutinin protein based on large-scale *ab initio* fragment molecular orbital calculations. J. Mol. Graph. Model..

[26] Anzaki S., Watanabe C., Fukuzawa K., Mochizuki Y., Tanaka S. (2014). Interaction energy analysis on specific binding of influenza virus hemagglutinin to avian and human sialosaccharide receptors: Importance of mutation-induced structural change. J. Mol. Graph. Model..

[27] Ohishi K., Suzuki R., Maruyama T., Romero A., Keith E.O. (2014). Host-virus specificity of the morbillivirus receptor, SLAM, in marine mammals: Risk assessment of infection based on three-dimensional models. New Approaches to the Study of Marine Mammals.

[28] Vongpunsawad S., Oezgun N., Braun W., Cattaneo R. (2004). Selectively receptor-blind measles viruses: Identification of residues necessary for SLAM- or CD46-induced fusion and their localization on a new hemagglutinin structural model. J. Virol..

[29] Masse N., Ainouze M., Néel B., Wild T.F., Buckland R., Langedijk J.P. (2004). Measles virus (MV) hemagglutinin: Evidence that attachment sites for MV receptors SLAM and CD46 overlap on the globular head. J. Virol..

[30] Leonard V.H., Sinn P.L., Hodge G., Miest T., Devaux P., Oezguen N., Braun W., McCray P.B., McChesney M.B., Cattaneo R. (2008). Measles virus blind to its epithelial cell receptor remains virulent in rhesus monkeys but cannot cross the airway epithelium and is not shed. J. Clin. Investig..

[31] Massé N., Barrett T., Muller C.P., Wild T.F., Buckland R. (2002). Identification of a second major site for CD46 binding in the hemagglutinin protein from a laboratory strain of measles virus (MV): Potential consequences for wild-type MV infection. J. Virol..

[32] Bluming A.Z., Ziegler J.L. (1971). Regression of Burkitt’s lymphoma in association with measles infection. Lancet.

[33] Taqi A.M., Abdurrahman M.B., Yakubu A.M., Fleming A.F. (1981). Regression of Hodgkin’s disease after measles. Lancet.

[34] Grote D., Russell S.J., Gornu T.I., Cattaneo R., Vile R., Polang G.A., Fielding A.K. (2001). Live attenuated measles virus induces regression of human lymphoma xenografts in immunodeficient mice. Blood.

[35] Peng K.W., Ahmann G.J., Pham L., Greipp P.R., Cattaneo R., Russell S.J. (2001). Systemic therapy of myeloma xenografts by an attenuated measles virus. Blood.

[36] Masaouel P., Opyrchal M., Dispenzieri A., Peng K.W., Federspiel M.J., Russell S.J., Galanis E. (2017). Clinical trials with oncolytic measles virus: Current status and future prospects. Curr. Cancer Drug Targets.

[37] Takano A., Ishikawa N., Nishino R., Masuda K., Yasui W., Inai K., Nishimura H., Ito H., Nakayama H., Miyagi Y. (2009). Identification of nectin-4 oncoprotein as a diagnostic and therapeutic target for lung cancer. Cancer Res..

[38] Derycke M.S., Pambuccian S.E., Gilks C.B., Kalloger S.E., Ghidouche A., Lopez M., Bliss R.L., Geller M.A., Argenta P.A., Harrington K.M. (2010). Nectin 4 overexpression in ovarian cancer tissues and serum: Potential role as a serum biomarker. Am. J. Clin. Pathol..

[39] Sugiyama T., Yoneda M., Kuraishi T., Hattori S., Inoue Y., Sato H., Kai C. (2013). Measles virus selectively blind to signaling lymphocyte activation molecule as a novel oncolytic virus for breast cancer treatment. Gene Ther..

[40] Fujiyuki A., Yoneda M., Amagi Y., Obayashi K., Ikeda F., Shoji K., Murakami Y., Sato H., Kai C. (2015). A measles virus selectively blind to signaling lymphocytic activation molecule shows anti-tumor activity against lung cancer cells. Oncotarget.

[41] Awano M., Fujiyuki A., Shoji K., Amagi Y., Murakami Y., Furukawa Y., Obayashi K., Sato H., Yoneda M., Kai C. (2016). Measles virus selectively blind to signaling lymphocyte activity molecule has oncolytic efficacy against nectin-4-expressing pancreatic cancer cells. Cancer Sci..

