# Supplementary material for: Computational Analysis of the Interaction Energies between Amino Acid Residues of the Measles Virus Hemagglutinin and Its Receptors
Source: Viruses. 2018 May 3;10(5):236. doi: 10.3390/v10050236 (PMC5977229; doi:10.3390/v10050236)
Supplement: Supplementary file 1 [file viruses-10-00236-s001.pdf]

Supplementary Material for

“Computational Analysis of the Interaction Energies between Amino Acid Residues of the Measles Virus Hemagglutinin and Its Receptors”

Fengqi Xu[1], Shigenori Tanaka[1*], Hirofumi Watanabe[2], Yasuhiro Shimane[3], Misako Iwasawa[4], Kazue Ohishi[5], Tadashi Maruyama[3]

[1] Department of Computational Science, Graduate School of System Informatics, Kobe University, 1-1, Rokkodai, Nada-ku, Kobe, Hyogo 657-8501, Japan

[2] Education Center on Computational Science and Engineering, Kobe University, 7-1-48, Minatojimaminamimachi, Chuo-ku, Kobe 650-0047, Japan

[3] Center for Marine Biosciences, Japan Agency for Marine-Earth Science and Technology, 2-15, Natsushima, Yokosuka, Kanagawa 237-0061, Japan

[4] Center for Earth Information Science and Technology, Japan Agency for Marine-Earth Science and Technology, 2-15, Natsushima, Yokosuka, Kanagawa 237-0061,Japan

[5] Faculty of Engineering, Tokyo Polytechnic University, 1583, Iiyama, Atsugi, Kanagawa 243-0297, Japan

*Correspondence: tanaka2@kobe-u.ac.jp; Tel: +81-78-803-6620

Table S1 shows the second and third most important amino-acid (a.a.) residues in (a) SLAM, (b) Nectin-4, and (c) CD46 receptors (designated as “2nd receptor a.a.” and “3rd receptor a.a.”, respectively) which have strongly attractive interactions with the higher ranked residues in MVH (second column in Table S1) shown in Table 2 in the main text. The 1st amino-acid (a.a.) residues which showed the highest IFIEs are shown in Table 2 in the main text. The “+” and “-” symbols in the parentheses after the residue names refer to the positively and negatively charged residues, respectively. The “n” symbol indicates that there is no residue in the receptor within the distance of 5 Å from the respective MVH residue. The “—” symbol indicates that the pertinent residue in the receptor shows the (small) repulsive interaction with the respective MVH residue.

Table S2 shows the effects of amino-acid (a.a.) substitution G546S of MVH on the distances and the IFIEs between the residues of the Chain D of CD46 and the respective corresponding residues of MVH.

Table S1. The second and third most important amino-acid (a.a.) residues in (a) SLAM, (b) Nectin-4, and (c) CD46 receptors (designated as "2nd receptor a.a." and "3rd receptor a.a.", respectively) which have strongly attractive interactions with the higher ranked residues in MVH (second column below) shown in Table 2 in the main text.

(a) SLAM					
Rank	MVH a.a.	2nd receptor a.a.	IFIE (kcal/mol)	3rd receptor a.a.	IFIE (kcal/mol)
1	Asp507(-)	Arg90(+)	-53.14	n	n
2	Asp505(-)	Ile78	-0.96	Leu92	-0.41
3	Arg533(+)	Ser80	-3.80	Ile62	-2.27
4	Arg556(+)	Ile126	-14.89	Asn125	-13.61
5	Asp530(-)	Ser80	-1.20	n	n
6	Glu503(-)	Leu92	-10.71	Asn76	-6.05
7	Arg195(+)	Gln129	-7.26	Val128	-2.94
8	Thr193	Phe131	-14.16	Gln129	-2.99
9	Phe552	Thr121	-6.48	Glu123	-4.07
10	Tyr551	Val128	-0.23	n	n
11	Tyr553	Val128	-2.56	Ile126	-1.88
12	Ser532	Lys77(+)	-3.37	Arg90(+)	-2.09
13	Phe483	Val74	-0.66	Ser73	—
14	Gly196	Ile126	—	Val128	—
15	Pro554	Thr121	—	His61	—
16	Tyr541	Arg130(+)	-1.96	Asn76	-0.38
17	Thr192	Arg130(+)	—	Gln129	—
18	Ile194	Val128	-2.19	Thr121	-0.75
19	Val534	His61	-0.66	Ser80	-0.42
20	Tyr543	Glu75(-)	-2.21	Asn76	—

(b) Nectin-4					
Rank	MVH a.a.	2nd receptor a.a.	IFIE (kcal/mol)	3rd receptor a.a.	IFIE (kcal/mol)
1	Arg547(+)	Phe106	-3.26	Gln107	-1.36
2	Ser550	Glu29(-)	-3.73	Gly1	—
3	Gln391	Lys54(+)	-3.16	n	n
4	Tyr543	Ala103	-5.21	Pro102	-2.27
5	Thr392	Tyr55	—	n	n
6	Leu500	Ser53	-2.16	Gly32	-2.13
7	Leu464	Ala103	-3.74	Ser105	-1.10
8	Phe483	Gly104	-2.54	Ser99	-2.12
9	Gly506	n	n	n	n
10	Ser548	Phe106	-2.09	Gly104	-0.39
11	Gly465	Gly104	—	Pro102	—
12	Gly388	Lys54(+)	-1.56	Gly56	-0.16
13	Tyr499	Tyr55	-0.32	n	n
14	Tyr524	Phe106	-1.21	Ser105	-0.59
15	Lys460(+)	n	n	n	n
16	Val485	His52	-0.63	Leu50	-0.04
17	Pro486	Leu50	-0.42	n	n
18	Ala463	n	n	n	n
19	Pro458	Pro102	-0.11	Phe101	—
20	Ile390	n	n	n	n

(c) CD46					
Rank	MVH a.a.	2nd receptor a.a.	IFIE (kcal/mol)	3rd receptor a.a.	IFIE (kcal/mol)
1	Lys477(+)	Gln75	-5.20	Ala76	—
2	Glu503(-)	Thr42	-7.76	Leu40	-2.59
3	Gly546	Val113	-3.38	Ala114	-1.51
4	Lys488(+)	Asn80	-0.59	Thr82	—
5	Tyr481	Tyr67	-3.85	Tyr83	-3.21
6	Glu471(-)	Tyr67	-21.12	Asp70(-)	—
7	His448	Tyr67	-2.08	Ile68	-1.38
8	Pro501	Leu40	-0.88	Ser56	-0.82
9	Phe483	Ile37	-2.13	Phe35	-0.73
10	Pro486	Gly81	-2.39	n	n
11	Tyr543	Tyr36	-2.94	Phe35	-1.31
12	Arg547(+)	Ser112	-9.34	Val113	—
13	Thr498	n	n	n	n
14	Val485	Tyr61	—	n	n
15	Tyr541	Tyr36	-1.49	Ile37	-1.12
16	His495	Gly81	—	n	n
17	Val451	Arg69(+)	-1.51	n	n
18	Pro545	Thr64	-0.90	Phe35	-0.59
19	Thr469	n	n	n	n
20	Leu462	Leu40	-0.55	n	n

The 1st amino-acid (a.a.) residues which showed the highest IFIEs are shown in Table 2 in the main text. The "+" and "-" symbols in the parentheses after the residue names refer to the positively and negatively charged residues, respectively. The "n" symbol indicates that there is no residue in the receptor within the distance of 5 Å from the respective MVH residue. The "—" symbol indicates that the pertinent residue in the receptor shows the (small) repulsive interaction with the respective MVH residue.

Table S2. Effects of amino-acid (a.a.) substitution G546S of MVH on the distances and IFIEs between the residues of the Chain D of CD46 and the respective corresponding residues of MVH.

	Before substitution			After substitution		
Residue (a.a.) of Chain D of CD46	MVH a.a.	Distance (Å)	IFIE (kcal/mol)	MVH a.a.	Distance (Å)	IFIE (kcal/mol)
Glu63	Pro545	2.4	-1.9	Pro545	2.4	-1.1
Glu63	Gly546	1.7	-27.2	Ser546	1.4	-24.5
Val113	Gly546	2.7	-3.4	Ser546	2.2	-4.4
Ser112	Gly546	4.6	-0.2	Ser546	4.1	-1.3
Glu63	Arg547	3.8	-45.4	Arg547	3.5	-63.0
Ser112	Arg547	2.4	-9.3	Arg547	2.1	-8.8
Val113	Arg547	3.5	1.9	Arg547	3.2	-2.0
Ala114	Arg547	4.6	3.5	Arg547	4.1	4.9
Lys32	Arg547	4.1	43.1	Arg547	4.6	37.3
